# Multi-user motion recognition using sEMG *via* discriminative canonical correlation analysis and adaptive dimensionality reduction

**DOI:** 10.3389/fnbot.2022.997134

**Published:** 2022-10-28

**Authors:** Jinqiang Wang, Dianguo Cao, Yang Li, Jiashuai Wang, Yuqiang Wu

**Affiliations:** School of Engineering, Qufu Normal University, Rizhao, China

**Keywords:** surface electromyography, discriminative canonical correlation analysis, adaptive dimensionality reduction, multi-user, motion recognition

## Abstract

The inability of new users to adapt quickly to the surface electromyography (sEMG) interface has greatly hindered the development of sEMG in the field of rehabilitation. This is due mainly to the large differences in sEMG signals produced by muscles when different people perform the same motion. To address this issue, a multi-user sEMG framework is proposed, using discriminative canonical correlation analysis and adaptive dimensionality reduction (ADR). The interface projects the feature sets for training users and new users into a low-dimensional uniform style space, overcoming the problem of individual differences in sEMG. The ADR method removes the redundant information in sEMG features and improves the accuracy of system motion recognition. The presented framework was validated on eight subjects with intact limbs, with an average recognition accuracy of 92.23% in 12 categories of upper-limb movements. In rehabilitation laboratory experiments, the average recognition rate reached 90.52%. The experimental results suggest that the framework offers a good solution to enable new rehabilitation users to adapt quickly to the sEMG interface.

## 1. Introduction

Surface electromyography (sEMG) signals are electrical signals that occur on the surface of human skin when the motor unit motion potential of a motor-associated muscle propagates along the muscle fibers (Vigotsky et al., 2018; Medved et al., 2020). These signals contain a wealth of information including muscle contraction force and joint torque (Disselhorst-Klug et al., 2009). In recent years, extensive research has been conducted into human–robot collaborative robots, teleoperated surgical robots, rehabilitation robots, wearable monitoring devices, and medical diagnosis based on sEMG signals (Ghassemi et al., 2019; Li et al., 2020; Luo et al., 2020a, 2021; Qi and Aliverti, 2020; Su et al., 2020, 2022; Qi et al., 2021). In particular, for stroke patients and patients with limb muscle injuries, a rehabilitation method in which the healthy side drives the affected side (an exoskeleton robot drives the limb on the affected side by recognizing the movements of the healthy side) can accelerate neurological remodeling and rehabilitation. However, people in these groups cannot provide a large dataset for training classifier models because of their limited physical fitness (Fang et al., 2020). Moreover, muscle strength, amount of subcutaneous fat, skin impedance, the fixed position of the electrodes, the degree of muscle fatigue, and limb posture all differ significantly among different users. All of this makes it difficult for new users to fit models trained on other users (Lobov et al., 2018).

Owing to these problems, there has been much research on various aspects of sEMG, including signal preprocessing, feature extraction, feature optimization, and classification (Elamvazuthi et al., 2015; Bi et al., 2019; Simao et al., 2019). For instance, Pan et al. (2018) developed a general sEMG interface for continuous prediction of coordinated movements between the palmar fingers and wrist flexion/extension. This model can be customized based on the musculoskeletal model of the individual user, and it can fit multiple users, including upper-limb amputees. User-generic musculoskeletal models capture generalized relationships between neuromuscular signals and human-generated limb movements. The successful application of this model reportedly relies on recording sEMG signals of specific muscles. This means that the patient must determine the exact location of the upper extremity muscles, which poses some challenges in practical applications for upper extremity rehabilitation patients. Xue et al. (2021) proposed a new framework called CCA-OT to handle the problem of multi-user gesture recognition. The CCA-OT framework reduces the differences in the distribution of the feature matrix. However, it was reported that the data distribution differed significantly between the training and testing sets of the framework. The framework incorporates a new feature dimension of 45 dimensions, so there is room for further optimization. Matsubara and Morimoto (2013) proposed a bilinear model comprising (i) user-related and (ii) motion-related linear factors; it extracts user-independent features from users and classifies them with a support vector machine (SVM) classifier, and the classification recognition rate indicated that the model outperformed non-multi-user methods. However, the dimensions of the style and content variables were chosen by trial and error, which added to the difficulty of the experiment, and the performance of the model was affected significantly by the electrode placement offset. Despite some progress, considerable challenges remain in applying these findings to clinical implementation (Samuel et al., 2019; Jarque-Bou et al., 2021).

Herein, a novel multi-user sEMG framework is proposed, based on discriminative canonical correlation analysis (DCCA) and adaptive dimensionality reduction (ADR), to reduce the variability of human sEMG, eliminate complex redundant information, improve recognition rates, and even reduce the training time required for classifier models. First, we propose an ADR optimization method based on the derived DCCA algorithm and design an ADR–DCCA architecture suitable for multiple-user action recognition. Then, the ADR–DCCA framework is verified to be superior to other CCA extensions through comparative experiments. Finally, the ADR-DCCA framework is used for an upper limb rehabilitation task in which the healthy side of the user drives the affected side. The contributions of this work can be summarized as follows.

- The DCCA algorithm is applied to the variability of human sEMG to project the views of two sEMGs of different users into a low-dimensional uniform style space. In this space, the intra-class correlation of the new features is guaranteed to be maximized and the inter-class correlation is minimized.

- The ADR optimization method is proposed; this is the main contribution of this work. This method selects the most suitable dimension for motion recognition from the new features, which effectively reduces the redundant information.

**Figure 1:**
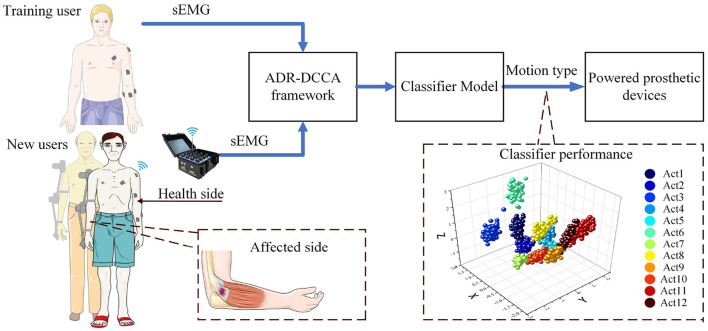
A multi-user health-side-driven rehabilitation system with an ADR-DCCA framework. The sEMG signals are collected from the healthy side arm of a new user and processed accordingly in the ADR-DCCA framework. The recognition is done by an upper limb exoskeleton robot.

The framework presented here can further extract the common information of training users and new users, effectively reduce personality information, and find the most suitable dimension for new users' action classification. New users can adapt to the sEMG interface using only a few sets of actions. In addition, the framework can be used to build an accurate model for a new user when two channels of the sEMG signal are missing. It is important for the patient to quickly obtain a model of the healthy side to drive the affected side. A multi-user contralateral-driven rehabilitation system is shown in Figure 1.

The structure of this paper is as follows. Section 2 describes the CCA and DCCA methods and then introduces the multi-user sEMG motion recognition framework. Section 3 describes the experimental data acquisition scheme. Section 4 shows the effectiveness of the proposed multi-user sEMG interface through multiple sets of comparative tests and analyses. Finally, Section 5 concludes the paper and discusses possible extensions and future directions.

## 2. Methods

CCA technique, which was introduced by Hotelling in 1936 (Hotelling, 1992), is a multivariate statistical method for studying the correlation between two sets of variables. It quantifies the association between two sets of variables and transforms the analysis of the correlation into an analysis between linear combinations of the two sets of variables. CCA has been widely used in the fields of data mining, machine learning, signal processing, biomedical engineering, healthcare data analysis, genetics, etc. However, to our knowledge, researchers have not yet addressed the problem of processing new feature dimensions after projection. Here, we develop an ADR-DCCA framework by combining the values of the fitness functions of SVM.

### 2.1. Theory of discriminative canonical correlation analysis

In our framework, an improved CCA method maps the sEMG features of different individuals into the same low-dimensional space to train a classifier model, which improves the applicability and generalization ability of the model to some extent. Considering the properties of the training user feature matrix and the new user calibration feature matrix, we define two column vectors, $X={\left(\begin{array}{c} X_{1}^{\left(1\right)},\cdots \;,X_{n}^{\left(1\right)},\cdots \cdots \;,X_{1}^{\left(c\right)},\cdots \;,X_{n}^{\left(c\right)} \end{array}\right)}^{T}$and $Y={\left(\begin{array}{c} Y_{1}^{\left(1\right)},\cdots \;,Y_{n}^{\left(1\right)},\cdots \cdots \;,Y_{1}^{\left(c\right)},\cdots \;,Y_{n}^{\left(c\right)} \end{array}\right)}^{T}$. Here, *c* is the motion category; *n* is the number of samples of the same type of motion; *p* and *q* are the number of features of a single sample in *X* and *Y*, respectively; and *X* and *Y* are standardized by column and expressed as


(1) $$\begin{array}{l} X=\left[\begin{array}{c} {X_{1}^{\left(1\right)}}^{T}\\ \vdots \\ {X_{n}^{\left(1\right)}}^{T}\\ \vdots \\ \vdots \\ {X_{1}^{\left(c\right)}}^{T}\\ \vdots \\ {X_{n}^{\left(c\right)}}^{T} \end{array}\right]=\left[\begin{array}{ccc} {X_{11}^{\left(1\right)}}^{T} & \cdots & {X_{1p}^{\left(1\right)}}^{T}\\ \vdots & \ddots & \vdots \\ {X_{n1}^{\left(1\right)}}^{T} & \cdots & {X_{np}^{\left(1\right)}}^{T}\\ \vdots & \vdots \\ \vdots & \vdots \\ {X_{11}^{\left(c\right)}}^{T} & \cdots & {X_{1p}^{\left(c\right)}}^{T}\\ \vdots & \ddots & \vdots \\ {X_{n1}^{\left(c\right)}}^{T} & \cdots & {X_{np}^{\left(c\right)}}^{T} \end{array}\right], \end{array}$$

(2) $$\begin{array}{l} Y=\left[\begin{array}{c} {Y_{1}^{\left(1\right)}}^{T}\\ \vdots \\ {Y_{n}^{\left(1\right)}}^{T}\\ \vdots \\ \vdots \\ {Y_{1}^{\left(c\right)}}^{T}\\ \vdots \\ {Y_{n}^{\left(c\right)}}^{T} \end{array}\right]=\left[\begin{array}{ccc} {Y_{11}^{\left(1\right)}}^{T} & \cdots & {Y_{1q}^{\left(1\right)}}^{T}\\ \vdots & \ddots & \vdots \\ {Y_{n1}^{\left(1\right)}}^{T} & \cdots & {Y_{nq}^{\left(1\right)}}^{T}\\ \vdots & \vdots \\ \vdots & \vdots \\ {Y_{11}^{\left(c\right)}}^{T} & \cdots & {Y_{1q}^{\left(c\right)}}^{T}\\ \vdots & \ddots & \vdots \\ {Y_{n1}^{\left(c\right)}}^{T} & \cdots & {Y_{nq}^{\left(c\right)}}^{T} \end{array}\right]. \end{array}$$

The basic idea of CCA is to find the variables $A={\left[a_{1},\cdots \;,a_{p}\right]}^{T}$ and $B={\left[b_{1},\cdots \;,b_{q}\right]}^{T}$ that maximize the correlation coefficient between the linear combinations *U*_*i*_ = *XA*_*i*_ and *V*_*i*_ = *YB*_*i*_, *A*_*i*_∈*A*, *B*_*i*_∈*B*, *U*_*i*_∈*U*, *V*_*i*_∈*V*(Hardoon et al., 2004; Sun et al., 2005). We choose the pair with the highest correlation coefficient in the linear combinations as the first set of canonical variables, the pair with the second-highest correlation coefficient as the second group of canonical variables, and so on until the correlation between *X* and *Y* is extracted (Sun et al., 2010); the correlation coefficient between canonical variables is called the canonical correlation coefficient. The optimization problem for *U*_*i*_ and *V*_*i*_ is given by


(3) $$\begin{array}{l} \arg\underset{A,B}{\max}\rho =corr\left(U,V\right)=\frac{cov\left(U,V\right)}{\sqrt{D\left(U\right)}\sqrt{D\left(V\right)}}. \end{array}$$

To simplify the expression, we let *cov*(*X, Y*) = *C*_12_, *cov*(*X, X*) = *C*_11_, *cov*(*Y, Y*) = *C*_22_, and we add the qualification $D\left(U\right)=A^{T}C_{11}A=1,D\left(V\right)=B^{T}C_{22}B=1$ to limit the occurrence of linear transformations of variables *A* and *B*. Then, the optimization function is


(4) $$\begin{array}{c} \;\arg\underset{A,B}{\max}\rho =corr\left(U,V\right)\\ \;=\frac{A^{T}C_{12}B}{\sqrt{A^{T}C_{11}A}\sqrt{B^{T}C_{22}B}}\\ \;=A^{T}C_{12}B\\ \text{such that }\left(\text{s}.\text{t}.\right)\;A^{T}C_{11}A=1,B^{T}C_{22}B=1. \end{array}$$

Using Lagrange multipliers, we convert the above optimization problem into a conditional extreme-value problem. By solving Equation (4), it is easy to obtain


(5) $$\begin{array}{l} {C_{11}}^{-1}C_{12}{C_{22}}^{-1}C_{21}A-\lambda^{2}A=0, \end{array}$$

(6) $$\begin{array}{l} {C_{22}}^{-1}C_{21}{C_{11}}^{-1}C_{12}B-\lambda^{2}B=0. \end{array}$$

From Equations (5) and (6), we know that ${C_{11}}^{-1}C_{12}{C_{22}}^{-1}C_{21}$ and ${C_{22}}^{-1}C_{21}{C_{11}}^{-1}C_{12}$ have the same eigenroots λ^2^, with *A* and *B* as the corresponding eigenvectors. The feature roots are arranged from largest to smallest, and the feature vectors are arranged in order of the corresponding feature roots. The feature vector that corresponds to the largest feature root is transformed *m* times according to the qualification to obtain the first set of *A*_1_, *B*_1_.

After that, we keep solving for the second set of canonical variables. Find variables *A*_2_ and *B*_2_ that maximize the correlation between *U*_2_ = *XA*_2_ and *V*_2_ = *YB*_2_ under the qualification $D\left(U_{2}\right)={A_{2}}^{T}C_{11}A_{2}=1,D\left(V_{2}\right)={B_{2}}^{T}C_{22}B_{2}=1$. The first pair of canonical variables has been extracted, so the second pair should be extracted without the information in the first pair (orthogonal to the first pair). We add a new qualification, $cov\left(U_{1},U_{2}\right)={A_{1}}^{2}C_{11}A_{2}=0,cov\left(V_{1},V_{2}\right)={B_{1}}^{2}C_{22}B_{2}=0$, with which we obtain


(7) $$\begin{array}{l} {C_{11}}^{-1}C_{12}{C_{22}}^{-1}C_{21}A_{2}-\lambda^{2}A_{2}=0, \end{array}$$

(8) $$\begin{array}{l} {C_{22}}^{-1}C_{21}{C_{11}}^{-1}C_{12}B_{2}-\lambda^{2}B_{2}=0. \end{array}$$

It is clear that the results *A*_2_, *B*_2_ of Equations (7) and (8) are the same *A, B* as those obtained from Equations (5) and (6). Therefore, the eigenvector corresponding to the next-largest eigenvalue is the one that we seek. Continuing in this way, it is easy to obtain the *r*th pair of eigenvectors, where *r* < min(*p, q*).

DCCA is based on CCA, which finds the largest intra-class correlation while minimizing the correlation across classes. DCCA can effectively improve the robustness of the generated features, thereby improving the performance of the whole system (Gatto and dos Santos, 2017). We define the vector$e_{nk}=\left[0_{1}^{\left(1\right)},\cdots \;,0_{n}^{\left(1\right)},\cdots \cdots \;,1_{1}^{\left(k\right)},\cdots \;,1_{n}^{\left(k\right)},\cdots \cdots \;,0_{1}^{\left(c\right)},\cdots \;,0_{n}^{\left(c\right)}\right]$, *k*∈(1, *c*), and then the intra-class correlation matrix is


(9) $$\begin{array}{l} C_{w}=\overset{c}{\underset{k=1}{\sum }}\overset{n}{\underset{i=1}{\sum }}\overset{n}{\underset{j=1}{\sum }}{X_{i}}^{\left(k\right)T}{Y_{j}}^{\left(k\right)}=\overset{c}{\underset{k=1}{\sum }}{\left(e_{nk}X\right)}^{T}\left(e_{nk}Y\right). \end{array}$$

We also define the row vector 1_*n*_ = [1, ⋯ , 1]_1 × (*c*×*n*)_; then, the inter-class correlation matrix is


(10) Cb=∑k1=1c∑k2=1k2≠k1c∑i=1n∑j=1nXi(k1)TYj(k2)     =∑k1=1c∑k2=1c∑i=1n∑j=1nXi(k1)TYj(k2)-∑k=1c∑i=1n∑j=1nXi(k)TYj(k)     =(1nX)T(1nY)-∑k=1cXTenkTenkY     =-∑k=1cXTenkTenkY.

As both the *X* and *Y* datasets are column normalized, we have ${\left(1_{n}X\right)}^{T}\left(1_{n}Y\right)=0$. DCCA can be described as solving


(11) $$\begin{array}{l} \underset{A,B}{\max}\left(A^{T}C_{w}B-A^{T}C_{b}B\right)=\underset{A,B}{\max}\left(\sum_{k=1}^{c}A^{T}X^{T}e_{nk}{}^{T}e_{nk}YB\right)\\ \text{ s}.\text{t}.\;A^{T}X^{T}XA=1,B^{T}Y^{T}YB=1. \end{array}$$

Given the specificity of *e*_*nk*_, it is easy to prove that


(12) $$\underset{A,B}{\max}\;\left(\sum_{k=1}^{c}A^{T}X^{T}e_{nk}^{T}e_{nk}YB\text{​}\right)=\underset{A,B}{\max}\;\left(A^{T}X^{T}\sum_{k=1}^{c}\left(e_{nk}{}^{T}e_{nk}\right)\;YB\text{​}\right).$$

Therefore, the optimization function can be written as


(13) $$\begin{array}{l} \;\underset{A,B}{\max}\;\left(A^{T}X^{T}c\sum_{k=1}^{c}\left(e_{nk}{}^{T}e_{nk}\right)\;YB\right)\\ \text{s}.\text{t}.\;A^{T}X^{T}XA=1,B^{T}Y^{T}YB=1, \end{array}$$

and using Lagrangian multipliers to tackle Equation (13) gives


(14) $$\begin{array}{l} \begin{array}{c} {C_{11}}^{-1}C_{w}{C_{22}}^{-1}{C_{w}}^{T}A-\lambda^{2}A=0,\\ {C_{22}}^{-1}{C_{w}}^{T}{C_{11}}^{-1}C_{w}B-\lambda^{2}B=0. \end{array} \end{array}$$

Solving this system of equations shows that ${C_{11}}^{-1}C_{w}{C_{22}}^{-1}{C_{w}}^{T}$ and ${C_{22}}^{-1}{C_{w}}^{T}{C_{11}}^{-1}C_{w}$ have the same characteristic root λ^2^ and that *A* and *B* are the corresponding eigenvectors, which is the same conclusion as that obtained with CCA.

### 2.2. Multi-user sEMG interface with ADR and DCCA

**Figure 2:**
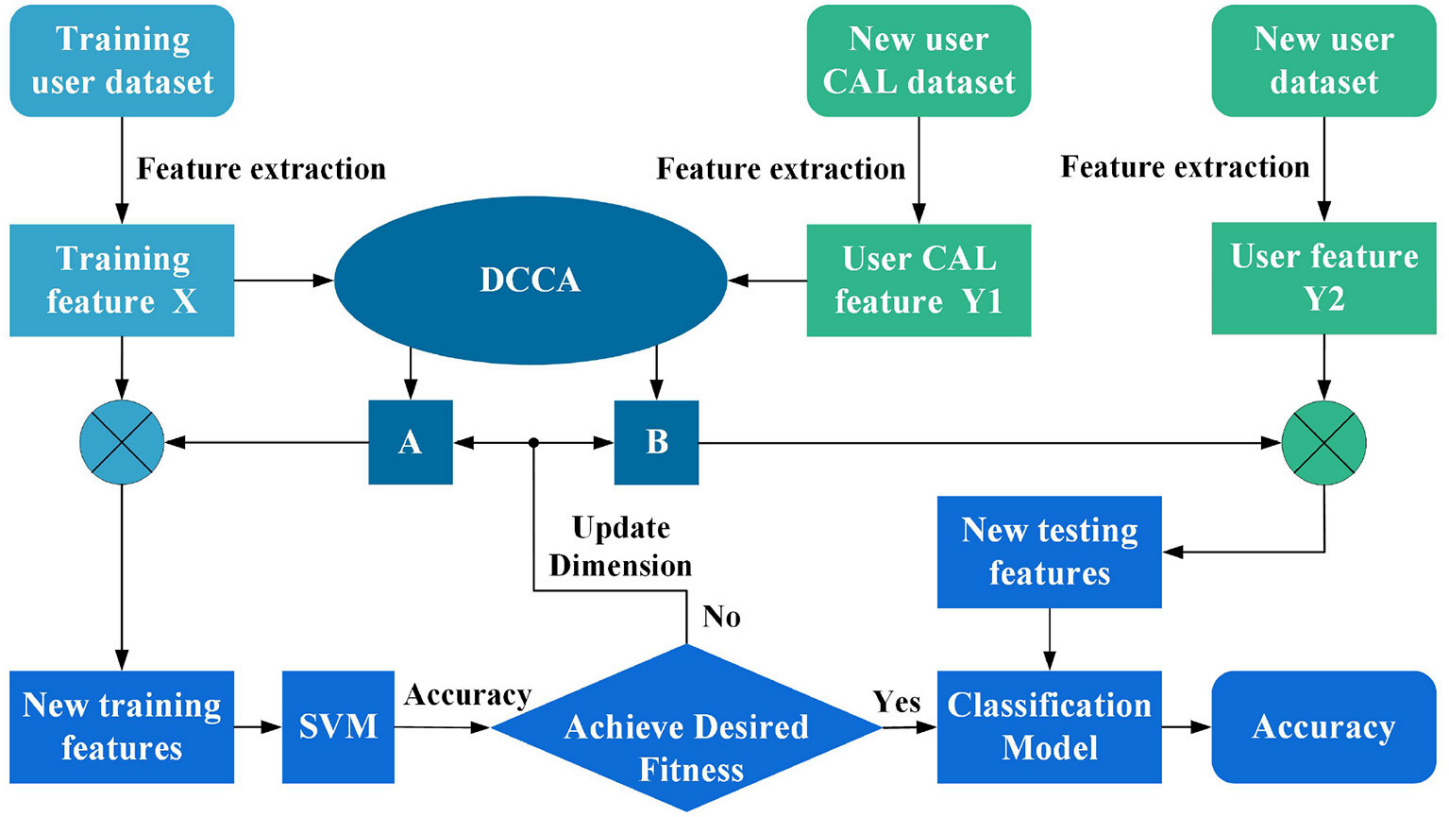
A multi-user surface electromyography (sEMG) motion-recognition framework was designed using discriminative canonical correlation analysis (DCCA) and adaptive dimensionality reduction (ADR).

The above derivation leads to the conclusion that DCCA seeks a pair of linear transformations or non-linear changes to maximize the intra-class correlation of two sets of features while ensuring the minimum inter-class correlation. The new features extracted by DCCA in different categories of the same feature set are statistically uncorrelated; thus, redundant information in different categories of features is eliminated. The proposed architecture is able to train classifier models with less data than traditional pattern recognition methods. The training data maintains a somewhat linear or non-linear relationship with the new test data, which helps new rehabilitation users to obtain accurate models faster. To the best of our knowledge, its use in developing an sEMG interface for multi-user is novel. We mapped the DCCA theory into the sEMG signal feature matrix and designed the framework shown in Figure 2, in which DCCA analyzes the training user feature matrix *X* and the new user calibration feature matrix *Y*1 (which is used to tune the parameters of the framework). The parameters that give strong correlation between the training user features and the new user features are obtained, which in this case are two independent matrices, namely, *A* and *B*. Then, new training features are obtained by *A* interacting with training user features *X*.

In this framework, the dimension of *A* is changed continuously according to the fitness function information provided by the SVM until the best model is found. Specifically, *a*_1_ denotes the first column of *A*, *a*_2_ denotes the first two columns of *A*, and so on; *a*_*l*_ (1 ≤ *l* ≤ *r*) denotes the first *l* columns of *A*. Thus, we can easily obtain the matrix *U*_*l*_ of different dimensions under the action of *a*_*l*_. By defining the SVM fitness function *f*(*t*), the value of the fitness function *f*(*U*_*l*_) can be obtained. The ADR problem can be expressed as an optimization problem for the following functions:


(15) $$\begin{array}{l} \arg\max\left\{f\left(U_{1}\right),f\left(U_{2}\right),\ldots ,f\left(U_{r}\right)\right\}. \end{array}$$

Finally, the new testing features are obtained based on the new user feature matrix *Y*2 and the matrix *B*_*l*_.

## 3. Signal acquisition

In the experiments described here, a customized Delsys wireless sEMG acquisition device was used for multichannel acquisition of sEMG signals. This device is a distributed contact electrode, which has the advantages of flexible placement and wireless convenience and is not affected by the lack of channels. The sampling rate of each sEMG channel of this device is 2,000 Hz with 16-bit resolution. The sEMG signals were displayed and stored in a PC client using sEMG-recorder software developed in-house, and the data for each action were saved in a separate .csv file.

**Figure 3:**
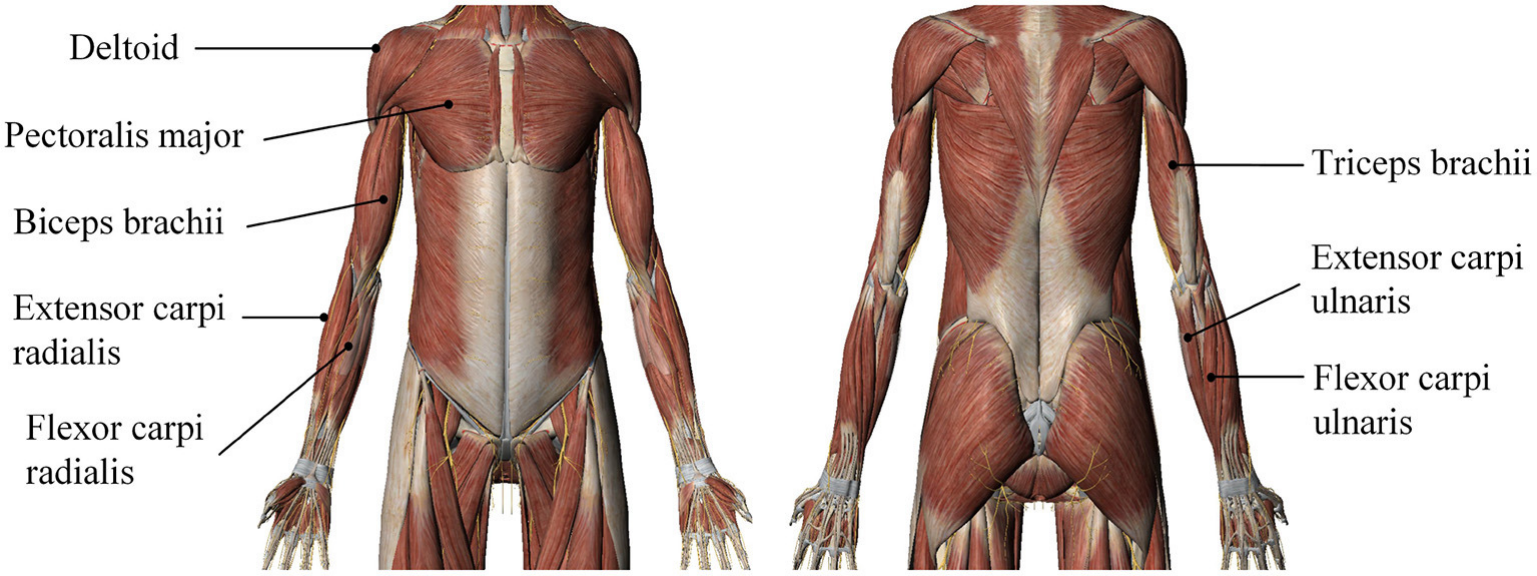
A map of precise locations of muscles for upper-limb movements.

The experiments involved eight participants (called N1 to N8) who were in good physical condition and had healthy limbs. The participants comprised seven men and one woman between the ages of 23 and 27 years; they participated voluntarily and were informed of the subject matter and signed an informed-consent form prior to participating in the experiments. The sEMG signals were recorded from the subject's right arm. Based on human physiology and upper-limb muscle distribution, we chose the deltoid (channel 1), pectoralis major (channel 2), biceps brachii (channel 3), triceps brachii (channel 4), flexor carpi radialis (channel 5), extensor carpi radialis (channel 6), extensor carpi ulnaris (channel 7), and flexor carpi ulnaris (channel 8). Position tracking was performed to determine the exact positions of the electrodes according to the criteria developed by the SENIAM (Surface ElectroMyoGraphy for the Non-Invasive Assessment of Muscles) European concerted action in the Biomedical Health and Research Program (BIOMED II) of the European Union (Hermens et al., 2000). The accurate muscle positioning is shown in Figure 3. Prior to data acquisition, the subjects' electrode pasting locations were cleared to prevent interference from hair; the skin at the relevant locations was then wiped with alcohol, and the electrodes were disinfected and finally pasted onto the prescribed muscle positions.

**Figure 4:**
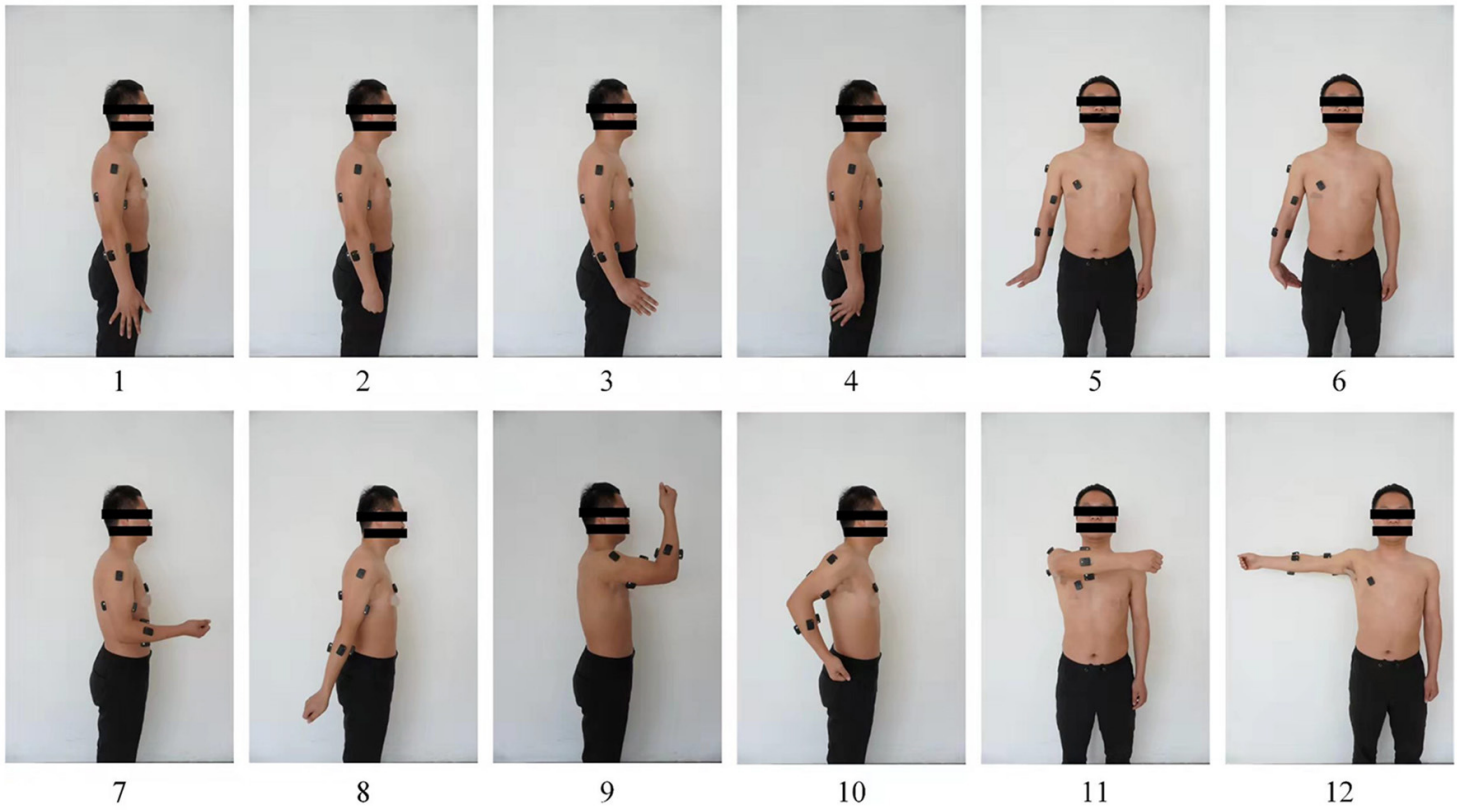
Upper-limb movements defined in present study: 1—palm extension; 2—fist clenching; 3—wrist abduction; 4—wrist adduction; 5—wrist extension; 6—wrist flexion; 7—elbow flexion; 8—elbow extension; 9—elbow flexion and shoulder flexion; 10—elbow flexion and shoulder extension; 11—shoulder horizontal adduction; 12—shoulder horizontal abduction.

During acquisition, each participant used no more than 80% of their maximum voluntary contraction to maintain control of their movements (Cheng et al., 2018). To ensure that the force used by the subject in performing the motion was within the required range, each subject was evaluated for force using a high-definition haptic device from QUANSER prior to data collection. During the recording process, the subject was asked to perform the following 12 upper-limb motor movements to the best of their ability with moderate constant force contractions: (i) palm extension, (ii) fist clenching, (iii) wrist abduction, (iv) wrist adduction, (v) wrist extension, (vi) wrist flexion, (vii) elbow flexion, (viii) elbow extension, (ix) elbow flexion and shoulder flexion, (x) elbow flexion and shoulder extension, (xi) shoulder horizontal adduction, and (xii) shoulder horizontal abduction (see Figure 4). When the subject heard a beep from the computer, they performed the specified movement, holding the final position for 3 s and then resting for 3 s before the next movement. Each subject completed the 12 movements 100 times, and in total 9,600 sets of experimental data were recorded from the eight participants. Considering that participants might become fatigued during sEMG collection, data were collected for no more than 5 min at a time.

## 4. Experiments and analysis

In this section, we describe how we evaluated the recognition capability of the proposed ADR-DCCA framework to show its feasibility. The preprocessing, feature extraction, and classification processes were carried out in the sEMG signal-processing pipeline.

In multichannel sEMG studies, preprocessing is necessary, mainly to reduce noise (Brunelli et al., 2015). In sEMG signals, this consists mainly of system noise, artifacts, industrial frequency interference, and channel cross-talk (De Luca et al., 2010). In the experiments, we used the wavelet algorithm, chose the sym5 wavelet function with five decomposition layers, and used henrsure to de-baseline and drift the sEMG signal of each channel. The signal was filtered with a fourth-order Butterworth filter to improve the signal-to-noise ratio and to keep the signal in the range of 20–500 Hz while removing external noise and artifacts.

Feature extraction is an important step in signal processing, as it reduces the amount of data while also extracting useful features into low-dimensional data. As EMG signals are known to suffer from lack of smoothness, windowing was performed on the sEMG signals after pre-processing (Ashraf et al., 2021). The window length was 300 samples with a time period of 150 ms, and the window shift was 50 samples with a time period of 25 ms (Luo et al., 2020b). After adding windows to the sEMG data, features including mean absolute value, root mean square, variance (Ahsan et al., 2011), maximum mean absolute value (MMAV), maximum root mean square (MRMS), maximum variance (MVAR) (MMAV, MRMS, and MVAR are improved features by the authors), fourth-order power spectral density, and power spectrum estimation (Zhang et al., 2011; Khushaba, 2014). The dimensionality of the extracted features was 64 features per motion, i.e., 8 channels × 8 features.

Based on the data gathered as described in Section 3, the experiments reported in this section used a fully separate design method for the training and testing sets. That is, one of the eight users was selected as a new user, and the remaining 7 people's data were treated as training user data. We call the process after selecting the new user a trial. There were eight trials in total. According to the seven training users' data, up to seven recognition rates can be determined for each new user. We calculated the average recognition rate for each trial. The above method was used for both Experiment 1 and Experiment 2. Experiment 1 involved (i) testing the present dataset using the CCA-OT framework proposed by Xue et al. (2021), (ii) testing the present dataset using the CCA framework proposed by Khushaba (2014), (iii) testing the present dataset after adding ADR optimization to the framework of (ii), and (iv) testing the present dataset using the proposed ADR-DCCA method. For Experiment 2, Experiment 1 was repeated but with two channels removed randomly from the user test set.

### 4.1. Experiment 1

#### 4.1.1. User-independent CCA-OT framework

One of the eight participants was selected as a new user, while the other seven were selected as training users. The tests were performed according to the CCA-OT analysis method described by Xue et al. (2021), and the recognition rates for the eight trials are given in Table 1.

**Table 1:** Experimental results for user-independent CCA-OT framework.

**Training user**	**Test user**							

	**N1**	**N2**	**N3**	**N4**	**N5**	**N6**	**N7**	**N8**
N1	N/A	83.78	88.47	88.69	93.96	95.71	88.85	96.00
N2	84.27	N/A	86.02	84.37	94.42	95.84	91.81	87.79
N3	87.20	85.01	N/A	93.76	89.93	93.56	91.12	91.23
N4	84.01	85.93	88.78	N/A	94.58	91.05	95.38	95.77
N5	85.80	86.66	86.00	90.04	N/A	91.89	82.85	85.67
N6	91.30	86.09	85.61	88.71	93.86	N/A	84.68	85.39
N7	90.65	90.89	90.01	86.29	88.19	93.24	N/A	92.66
N8	93.96	84.20	85.13	87.02	95.04	88.07	85.71	N/A
Average accuracy	88.17	86.08	87.15	88.41	92.85	92.76	88.63	90.65

#### 4.1.2. User-independent CCA-without-ADR framework

Testing was carried out according to the CCA method described by Khushaba (2014), which reduces the differences in sEMG signal properties when the same motion is performed by different people. The recognition rates for the eight trials are given in Table 2.

**Table 2:** Experimental results for user-independent CCA-without-ADR framework.

**Training user**	**Test user**							

	**N1**	**N2**	**N3**	**N4**	**N5**	**N6**	**N7**	**N8**
N1	N/A	82.93	89.02	87.11	95.84	95.78	87.74	95.11
N2	85.10	N/A	84.70	82.63	89.14	95.16	88.05	89.08
N3	88.30	84.93	N/A	96.00	89.11	90.74	89.22	90.10
N4	81.32	84.58	87.43	N/A	89.68	90.12	92.80	93.42
N5	79.70	85.41	81.38	90.94	N/A	87.21	80.56	86.81
N6	89.57	89.43	85.99	84.67	92.12	N/A	82.41	85.35
N7	87.30	88.19	88.53	88.50	84.19	88.67	N/A	94.51
N8	90.20	82.46	84.22	87.27	95.07	83.49	85.77	N/A
Average accuracy	85.93	85.42	85.90	88.16	90.73	90.17	86.65	90.63

#### 4.1.3. User-independent CCA-with-ADR framework

Following the CCA method described by Khushaba (2014), we performed ADR optimization of the framework. The ADR optimization method chooses the most suitable reconstructed features for user classification. The recognition rates for the eight trials are given in Table 3.

**Table 3:** Experimental results for user-independent CCA-with-ADR framework.

**Training user**	**Test user**							

	**N1**	**N2**	**N3**	**N4**	**N5**	**N6**	**N7**	**N8**
N1	N/A	86.75	89.17	88.08	95.75	95.83	88.67	96.75
N2	86.00	N/A	85.17	85.00	88.83	95.42	90.08	88.50
N3	89.08	85.00	N/A	96.00	90.58	93.83	93.08	90.92
N4	83.67	87.00	90.17	N/A	93.75	91.92	94.83	94.92
N5	84.58	85.08	85.08	91.33	N/A	91.67	84.67	86.92
N6	92.58	89.08	86.25	88.67	95.92	N/A	87.08	85.08
N7	89.75	88.58	82.83	88.33	86.75	93.00	N/A	93.58
N8	93.58	85.08	87.25	86.33	95.58	87.67	85.67	N/A
Average accuracy	88.46	86.65	86.56	89.11	92.45	92.76	89.15	90.95

#### 4.1.4. User-independent DCCA-with-ADR framework

Using the method proposed herein, data from the training users and new user were fed into the ADR-DCCA framework. The user characteristics were rebuilt into low-dimensional features independent of individual styles *via* parameter pair optimization, and the user features overcame individual differences. This framework has the advantages of that of Khushaba (2014) while also minimizing the inter-class correlation of different motion features and adaptively selecting new feature dimensions that are most beneficial to the classification performance; this reduces classifier training time and improves classification accuracy. The recognition rates for the eight trials are given in Table 4.

**Table 4:** Experimental results for user-independent DCCA-with-ADR framework.

**Training user**	**Test user**							

	**N1**	**N2**	**N3**	**N4**	**N5**	**N6**	**N7**	**N8**
N1	N/A	89.88	92.42	91.67	96.42	98.25	90.67	97.00
N2	88.75	N/A	86.50	89.58	89.08	94.58	93.33	92.50
N3	90.33	91.50	N/A	96.67	97.67	96.67	95.42	91.50
N4	87.25	90.67	94.42	N/A	93.00	95.75	96.17	94.00
N5	87.33	87.92	88.50	92.17	N/A	96.67	91.17	89.50
N6	93.17	90.28	86.58	91.00	96.67	N/A	88.67	91.33
N7	89.50	92.58	88.33	94.00	89.17	97.83	N/A	93.75
N8	94.67	89.67	90.92	93.17	96.58	91.08	90.85	N/A
Average accuracy	90.14	90.36	89.67	92.61	94.08	95.83	92.33	92.80

**Figure 5:**
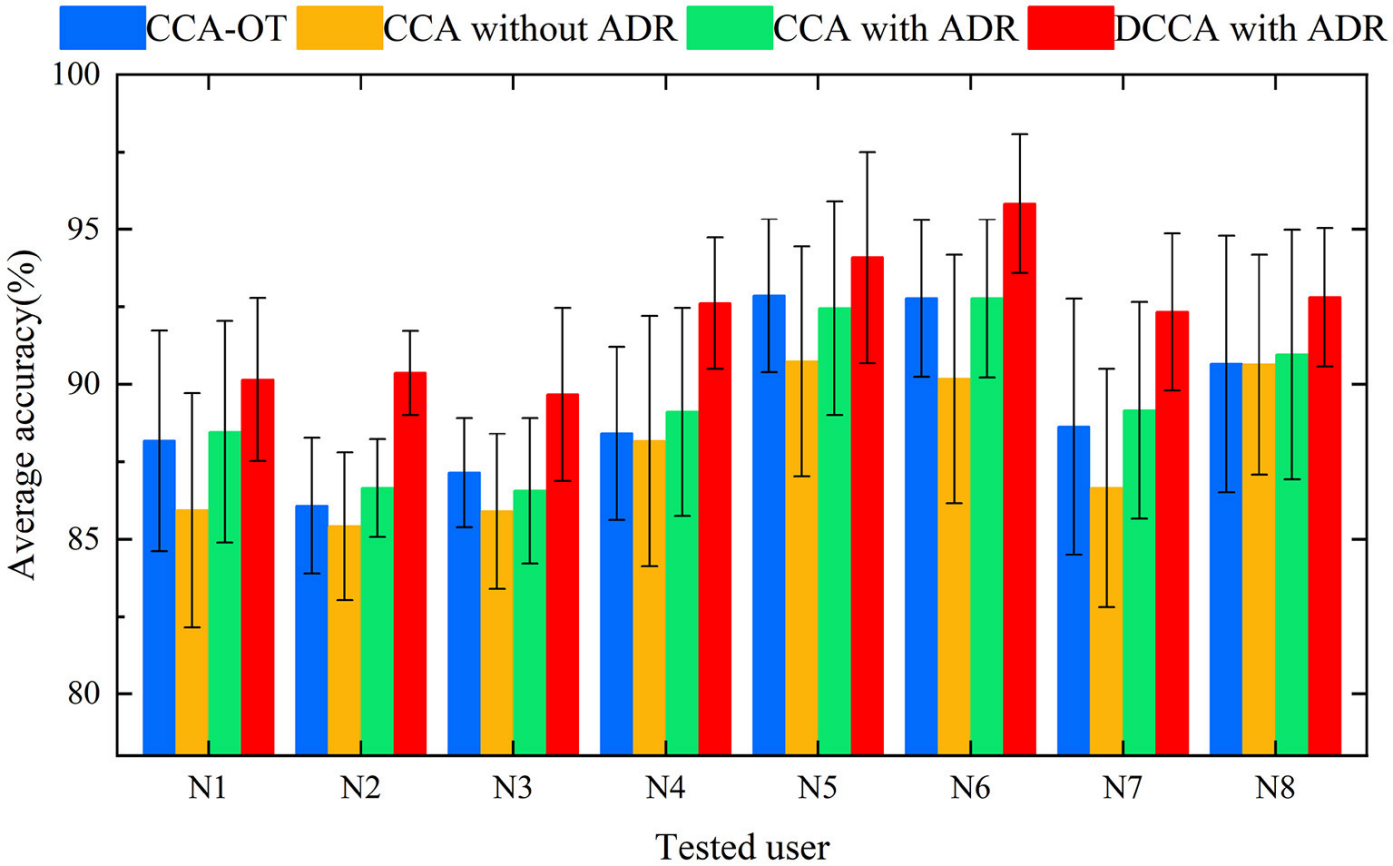
Histogram of average accuracies of 12 campaigns classified by four methods for eight users.

In Experiment 1, the dataset used in this study was tested with four methods, respectively. For each method, results were recorded for eight trials denoted N1 to N8. For the different methods, we plotted the mean values of each trial as histograms. In Figure 5, histograms of the same color show the average recognition accuracy for each test user with the same method, and error bars represent the standard deviation of this user's motion recognition rate on different training sets.

### 4.2. Experiment 2

**Figure 6:**
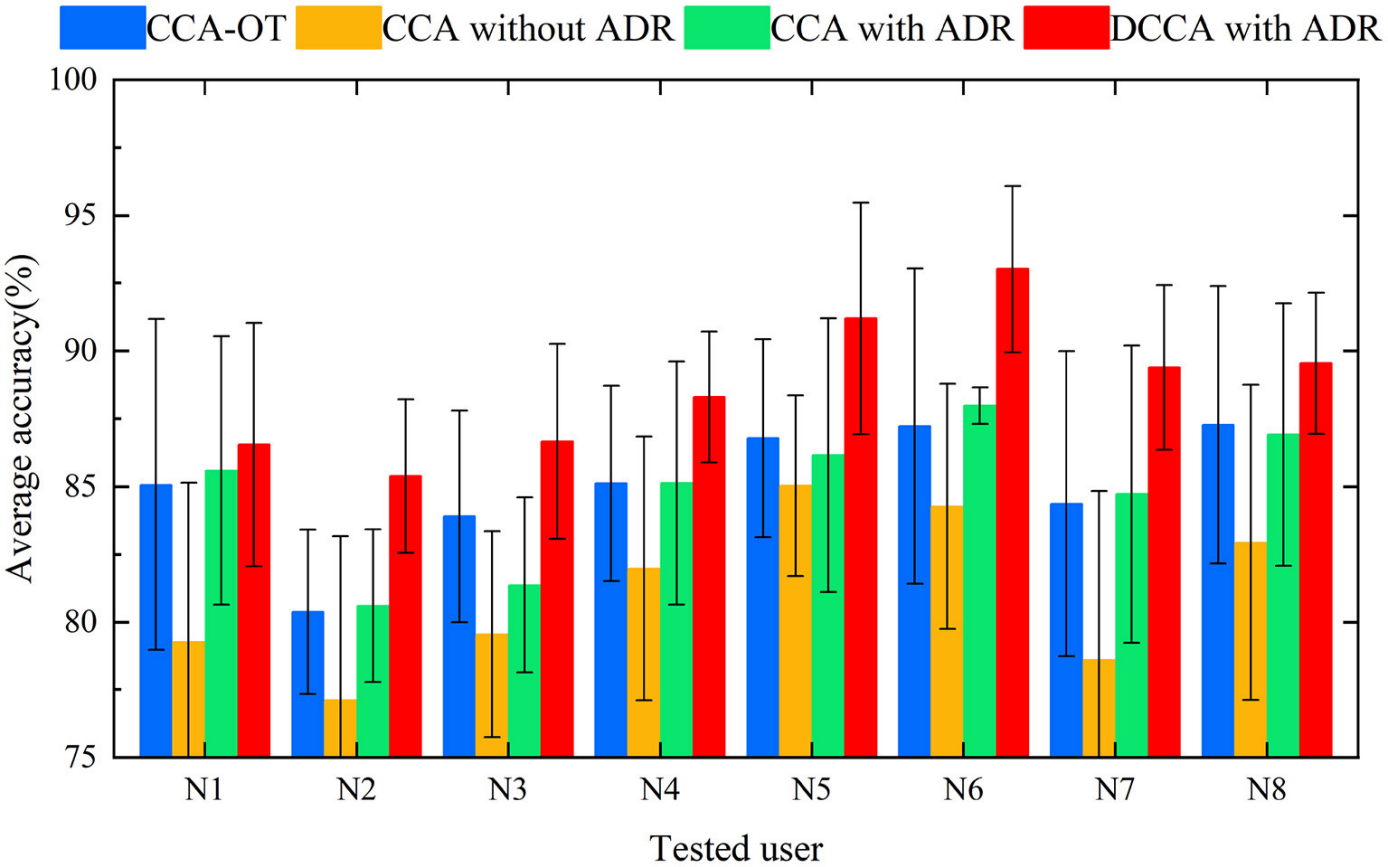
Histogram of average accuracies of 12 campaigns classified by four methods for eight users (randomly reducing the user test set by two channels).

The sEMG data of all test users were reduced randomly by two channels and then tested again as in Experiment 1 to verify the robustness of the method proposed herein. Tables 5–8 record the recognition results of eight trials (N1 to N8) for each method under 4 methods, and the average recognition rate of each trial was calculated. As in Experiment 1, for the different methods we plotted the average result of each test as a histogram. In Figure 6, histograms in the same color show the average recognition accuracy for each test user under the same method, and error bars represent the standard deviation of this user's motion recognition rate on different training sets.

**Table 5:** Experimental results for user-independent CCA-OT framework (the sEMG data of all test users were reduced randomly by two channels).

**Training user**	**Test user**							

	**N1**	**N2**	**N3**	**N4**	**N5**	**N6**	**N7**	**N8**
N1	N/A	74.87	83.91	79.03	92.82	96.48	84.59	96.70
N2	78.62	N/A	84.59	82.55	90.76	93.68	82.87	84.93
N3	81.24	78.70	N/A	89.26	81.94	89.07	90.12	90.03
N4	84.88	83.41	88.72	N/A	87.22	86.23	94.37	90.76
N5	76.22	78.16	85.80	89.99	N/A	82.55	80.80	80.43
N6	91.04	84.02	79.29	85.42	84.18	N/A	76.33	84.47
N7	90.70	82.06	87.70	83.08	83.58	83.71	N/A	83.58
N8	92.72	81.39	77.31	86.47	86.89	78.86	81.41	N/A
Average accuracy	85.06	80.37	83.90	85.12	86.77	87.22	84.36	87.27

**Table 6:** Experimental results for user-independent CCA-without-ADR framework (the sEMG data of all test users were reduced randomly by two channels).

**Training user**	**Test user**							

	**N1**	**N2**	**N3**	**N4**	**N5**	**N6**	**N7**	**N8**
N1	N/A	78.78	80.61	77.15	91.84	82.27	76.09	89.95
N2	76.96	N/A	73.67	74.97	83.23	89.58	86.88	78.47
N3	86.77	75.31	N/A	90.22	85.58	89.97	84.89	81.87
N4	79.37	77.05	78.52	N/A	85.68	86.16	84.44	88.16
N5	66.61	71.64	81.65	83.21	N/A	76.07	69.42	76.59
N6	81.56	90.19	85.79	85.42	80.04	N/A	74.83	75.71
N7	81.20	76.70	75.32	79.02	85.18	81.58	N/A	89.77
N8	82.45	70.05	81.29	83.79	83.64	84.21	73.58	N/A
Average accuracy	79.28	77.10	79.55	81.97	85.02	84.26	78.59	82.93

**Table 7:** Experimental results for user-independent CCA-with-ADR framework (the sEMG data of all test users were reduced randomly by two channels).

**Training user**	**Test user**							

	**N1**	**N2**	**N3**	**N4**	**N5**	**N6**	**N7**	**N8**
N1	N/A	85.75	83.82	78.84	91.59	87.52	85.68	89.55
N2	86.81	N/A	76.21	78.52	82.41	88.51	90.61	80.96
N3	87.11	81.54	N/A	91.84	81.74	88.67	85.73	88.27
N4	77.45	77.25	85.35	N/A	90.47	87.72	92.27	94.21
N5	81.00	79.76	82.74	85.81	N/A	86.70	77.85	80.71
N6	83.77	82.52	83.95	87.69	90.58	N/A	84.47	83.67
N7	89.72	80.36	79.85	86.23	77.75	88.70	N/A	91.01
N8	93.26	77.05	77.62	86.99	88.51	88.05	76.42	N/A
Average accuracy	85.59	80.60	81.36	85.13	86.15	87.98	84.72	86.91

**Table 8:** Experimental results for user-independent DCCA-with-ADR framework (the sEMG data of all test users were reduced randomly by two channels).

**Training user**	**Test user**							

	**N1**	**N2**	**N3**	**N4**	**N5**	**N6**	**N7**	**N8**
N1	N/A	84.78	87.49	85.37	94.25	95.72	90.25	91.86
N2	84.23	N/A	83.97	84.17	87.19	90.63	92.83	85.57
N3	90.57	87.74	N/A	89.49	95.39	92.80	89.73	91.96
N4	79.26	82.99	93.07	N/A	93.51	93.71	93.69	91.81
N5	83.49	81.50	82.69	90.32	N/A	95.60	88.41	85.82
N6	88.99	88.78	86.84	87.92	95.05	N/A	85.54	89.08
N7	85.65	88.84	82.75	89.83	83.30	95.79	N/A	90.65
N8	93.60	83.04	89.81	90.97	89.65	86.83	85.23	N/A
Average accuracy	86.54	85.38	86.66	88.30	91.19	93.01	89.38	89.54

### 4.3. Results and discussion

For this subsection, we performed preprocessing and feature extraction and extracted a feature set of 64 features per movement ([6 time-domain features + 2 frequency-domain features] × 8 channels). For the classification, we chose the well-known SVM LIBSVM (Chang and Lin, 2011), as Khushaba (2014) reported that a global SVM performs better in this type of action recognition. In our experimental approach, the training and calibration sets were completely independent, i.e., the training user and the new user were different individuals, and the experimental results were obtained through multiple cross-validations. Khushaba (2014) and Xue et al. (2021) performed CCA (i) between a training feature matrix and an expert feature matrix and (ii) between a test feature matrix and an expert feature matrix. In the present framework, DCCA is used to directly extract the correlation between the training user feature matrix and the new user feature matrix, thereby eliminating the intermediate link. The correlation between the training feature matrix and the test feature matrix is enhanced, and the distribution differences between classes are strengthened. The adaptive dimensionality-reduction method eliminates the redundant information in the high-dimensional features and retains the low-dimensional features that are highly correlated between the new user and the training users. High classification accuracy and good robustness were achieved in classifying 12 categories of upper-limb movements in eight subjects. In Experiment 1, the motion recognition rate of the ADR-DCCA framework proposed in this paper was 92.23; this was 2.89, 4.28, and 2.72 higher than the average recognition rates of the CCA-OT framework, the conventional CCA framework, and the ADR-CCA framework, respectively. In particular, compared with the 26–126 dimensions in the technique described by Khushaba (2014), we used 5–8 dimensions of features that were obtained by fusing 64 dimensions of features, thereby achieving higher accuracy in motion recognition. In Experiment 2, the identification accuracies of the CCA-OT framework, the conventional CCA framework, the ADR-CCA framework, and the ADR-DCCA framework were 85.01, 81.09, 84.81, and 88.75%, respectively. The experimental results show that the framework described herein maintains high classification accuracy even after being deprived of data from two channels, which indicates that the framework is robust. Moreover, the accuracy of motion classification was maintained at 88.75% in the test involving the random removal of two channels, despite the poor performance of the subjects compared with the full-channel subjects. The random channel deletion feature in the proposed framework provides a possible solution for the rehabilitation of muscle-deficient patients and motion recognition of missing channel users.

**Figure 7:**
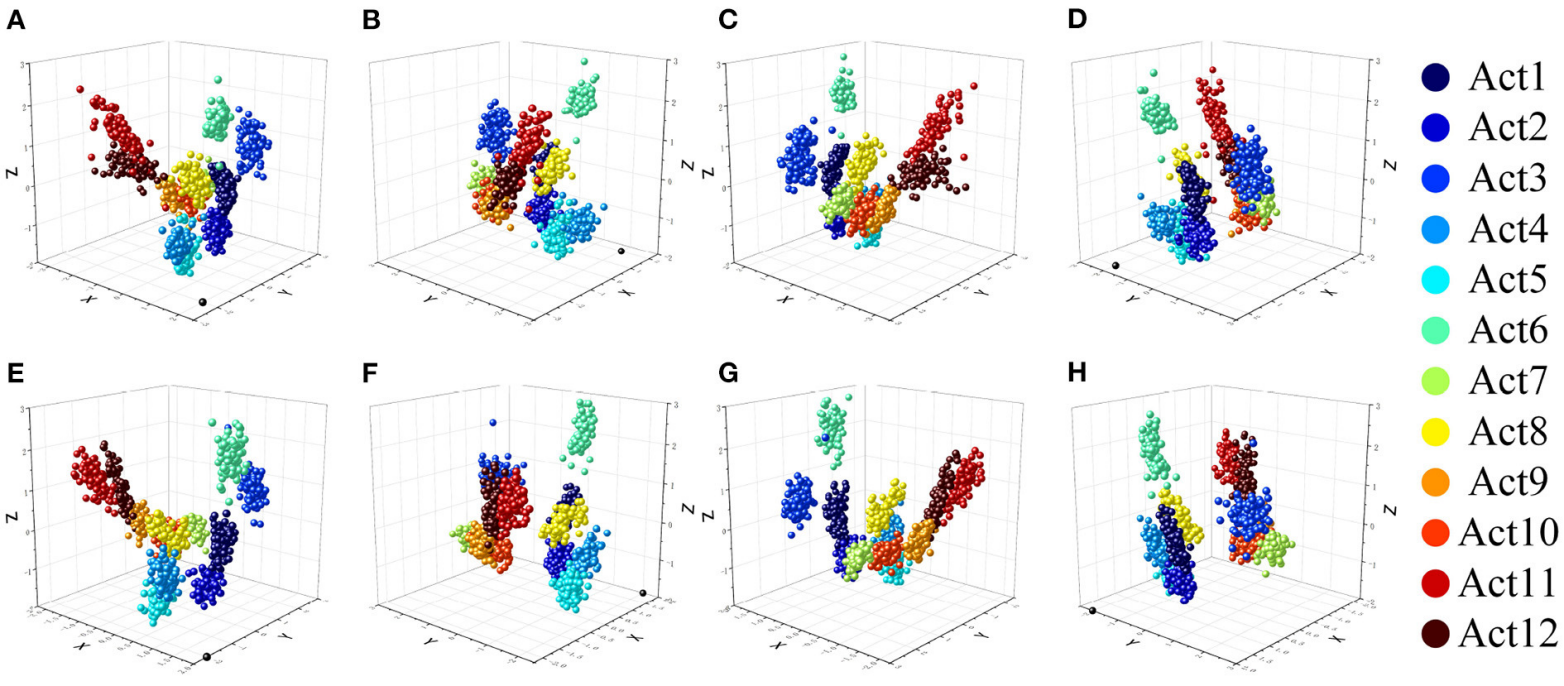
Three-dimensional scatter plots of three new features reconstructed using the CCA framework **(A–D)** and DCCA framework **(E–H)**. The 12 different-colored balls represent the different motions of the new user (the black ball at the bottom is the coordinate rotation mark).

The training dataset and calibration dataset were passed through the CCA and DCCA algorithms, respectively, to obtain two sets of parameters *V*. These two sets of *V* were used to construct each user's test feature matrix. After projection, two new sets of test feature matrices were obtained. The data in the top three columns of the most favorably identified features were plotted as a three-dimensional scatter plot. Figures 7A–D show the post-projection features observed by the CCA method from four directions, and Figures 7E–H show the same for the DCCA method. Compared with the plots for the CCA method, those for the DCCA method have more cohesive color blocks more cohesive and show lower intra-class variance while maintaining good inter-class separability, which is beneficial to classification performance. The results of the three-dimensional scatter plots also verify our hypothesis on the performance of DCCA.

**Figure 8:**
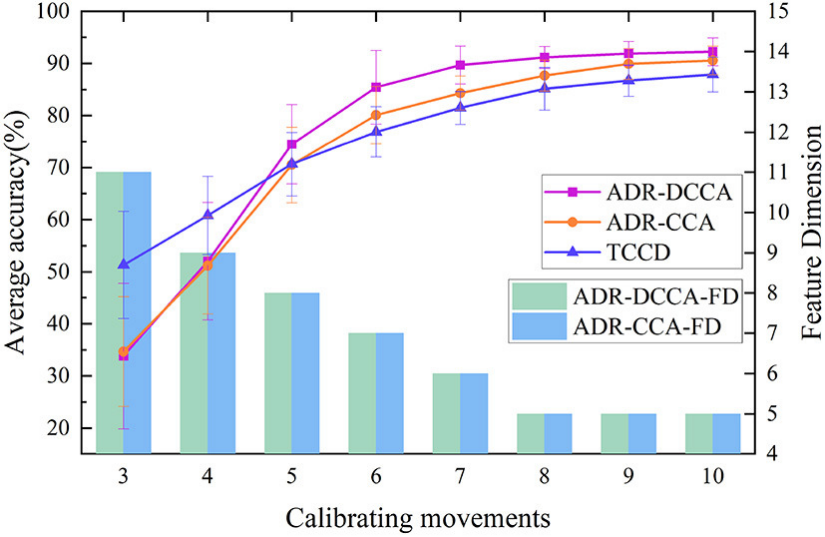
Relationships between sample size and classification accuracy and between sample size and new feature dimensions. ADR-DCCA and ADR-CCA denote the classification accuracy of the ADR-DCCA architecture and ADR-CCA with different numbers of calibration sets, respectively. ADR-DCCA-FD and ADR-CCA-FD are the corresponding feature dimensions under classification accuracy. TCCD indicates the classification accuracy when training the classifier model directly with the calibration dataset.

We also analyzed how the number of samples in the calibration process affects the classification accuracy, as well as the direct relationship among the number of samples and the new feature dimension. In this case, we tested the performance of the ADR-CCA and ADR-DCCA frameworks by selecting a certain number of samples from the calibration dataset. As shown in Figure 8, the accuracy of the classification significantly improved and the dimensionality of the features effectively decreased as the size of the calibration dataset increased. In addition, we performed experiments in which the classifier was trained directly with calibration data, where both calibration and test data were obtained from the same human body. Figure 8 shows the recognition results obtained when training the classifier with the calibration data in these experiments. Directly training the classifier with the calibration data using 3–4 sets of calibration data clearly resulted in better performance compared with the CCA method. Unfortunately, the classification performance of all three methods did not meet the application requirements. However, the proposed method ADR-DCCA method had the highest classification accuracy at 5–10 sets of calibration data. Smaller calibration datasets were chosen because this study was intended to target specific groups such as rehabilitation patients. Here, 3–10 sets of corrected data were selected for testing, and a good classification effect could be achieved with eight sets. The experimental results indicate that the ADR-DCCA framework has advantages over other CCA extension frameworks for multi-user sEMG interfaces.

## 5. Conclusion

In order to solve the problem of stroke patients or patients with limb muscle injuries being unable to quickly obtain a suitable contralateral model for rehabilitation training, a suitable multi-user sEMG motion-recognition framework is presented here. First, the training dataset and the new user calibration dataset are projected into the same style space. New features for training users and new users are recreated based on the parameters obtained after the projection. Second, the dimensionality of the new features is optimized iteratively using an adaptive dimensionality-reduction optimization method to obtain the dimensionality of the system features that are most suitable for motion recognition. Finally, a model is constructed that is suitable for new users and shows good classification performance in user test set classification. The experimental results indicate that the DCCA algorithm is effective at extracting the relevant information between the new user test set and the training user feature set, and that the ADR method effectively reduces the redundant information between the two datasets. These are both important factors in the improvement in motion recognition performance obtained with the framework. This framework also better addresses the problem of large differences in muscle-generated sEMG signals when different people perform the same movement and enables new users to engage in contralateral-driven rehabilitation patterns more quickly. The accuracy of motion classification was maintained at 88.75% in the test involving the random removal of two channels, despite the poor performance of the subjects compared with the full-channel subjects. The random channel deletion feature in the proposed framework provides a possible solution for the rehabilitation of muscle-deficient patients and motion recognition of missing channel users. Finally, we tested the proposed method in the rehabilitation laboratory, and the accuracy of action recognition reached 90.52%. In conclusion, our proposed ADR-DCCA method has important potential applications in the field of rehabilitation.

Although this work implemented sEMG-based action recognition for rehabilitation movements of upper limb exoskeleton robots and glove robots, the experiments only considered human variation and channel loss in sEMG action recognition. In fact, there are many sources of uncertainty in sEMG action recognition, including fatigue differences and electromagnetic interference. In addition, the sEMG-based information is limited. In future work, we will continue to improve the proposed framework and integrate EEG signals and acceleration-sensing information to enable it to cope with more non-ideal situations, thereby improving the comprehensive performance of action recognition.

## Data availability statement

The raw data supporting the conclusions of this article will be made available by the authors, without undue reservation.

## Ethics statement

The studies involving human participants were reviewed and approved by Qufu Normal University Biomedical Ethics Committee. The patients/participants provided their written informed consent to participate in this study. The animal study was reviewed and approved by Qufu Normal University Biomedical Ethics Committee. Written informed consent was obtained from the individual(s) for the publication of any potentially identifiable images or data included in this article.

## Author contributions

JW conceived the study, designed and conducted the experiments, performed the data analysis, and drafted and revised the manuscript. DC and YW provided guidance on the algorithms in the study and analyzed the experimental results. All authors contributed to manuscript revision and read and approved the submitted version.

## Funding

This work was partially supported by the National Natural Science Foundation of China (Grant 62073187), the Major Scientific and Technological Innovation Project in Shandong Province (Grant 2019JZZY011111), and the Natural Science Foundation of Shandong Province (Grant ZR2022MF236).

## Conflict of interest

The authors declare that the research was conducted in the absence of any commercial or financial relationships that could be construed as a potential conflict of interest.

## Publisher's note

All claims expressed in this article are solely those of the authors and do not necessarily represent those of their affiliated organizations, or those of the publisher, the editors and the reviewers. Any product that may be evaluated in this article, or claim that may be made by its manufacturer, is not guaranteed or endorsed by the publisher.
